# Editorial: Stress-Related Diseases and Dysfunctions

**DOI:** 10.3389/fphys.2022.896842

**Published:** 2022-04-12

**Authors:** Carlos Henrique Xavier, Rodrigo Cunha Alvim de Menezes, Deoclécio Alves Chianca, Marco Antônio Peliky Fontes, Carlos César Crestani

**Affiliations:** ^1^ Systems Neurobiology Laboratory, Institute of Biological Sciences, Federal University of Goiás, Goiânia, Brazil; ^2^ Department of Biological Sciences, Institute of Biological Sciences, Federal University of Ouro Preto, Ouro Preto, Brazil; ^3^ Department of Physiology and Biophysics, Institute of Biological Sciences, Federal University of Minas Gerais, Belo Horizonte, Brazil; ^4^ Department of Drugs and Medicines, School of Pharmaceutical Sciences, São Paulo State University (UNESP), Araraquara, Brazil

**Keywords:** stress, aversion, defense reactions, Physiology, Pathophysiology

Stress is a term that was firstly coined by Hans Hugo Bruno Selye, aimed at referring to environmental aspects challenging the balance of physiological functions (Selye, 1936). The noxious and non-noxious aversive situations that are perceived as stressful trigger a set of synchronically organized responses with specific and non-specific components (Shin and Liberzon, 2010; Sterling, 2012; McEwen and Akil, 2020), known as defense reactions. These reactive settings, capable of increasing the chances of surviving while facing stressful events, include behavioral, neuroendocrine, respiratory, metabolic/thermogenic, hydroelectrolytic, cardiocirculatory and autonomically-mediated adjustments of different tissues. Altogether, these strategic functional responses prepare the body for such defense (Dampney, 2015; Crestani, 2016; Herman et al., 2020; Herman, 2022) and shall be absent when the perceived risk and aversion are over. However, in some cases, brain maladaptation to stress can generate inadequate responses to environmental challenges manifested either as hyperreactivity or as perpetuation in the recruitment of physiological adjustments, acting as a risk factor for dysfunctions and diseases (VanItallie, 2002; Kivimäki and Kawachi, 2015; Lagraauw et al., 2015; Roth et al., 2020). Notwithstanding, the mechanistic features underlying different pathophysiological processes triggered from stressful situations are poorly understood.

Global epidemiological data show that the current knowledge on the mechanisms involved in the stress-related diseases and the therapeutic arsenal against these diseases need to be broadened, in order to pave ways for novel and efficient therapeutic approaches interfering directly with the morbidity and mortality. The understanding that environmental situations are able to impair mental health resulting in associated diseases was recently strengthened during the COVID-19 pandemic, in which neuropsychiatric manifestations remarkably coincided with increases in morbi-mortality related to non-infectious diseases (Wessler et al., 2020; COVID research, 2021; Deng et al., 2021; Nalbandian et al., 2021). Since the need for a better understanding on stress-evoked mechanisms resulting in diseases remains indeed pertinent, this Research Topic was dedicated to collect high quality science from cutting-edge research unraveling details on mechanisms involved in the dysfunctions and diseases related to acute and chronic stress exposure, in humans and in different experimental models. In this Research Topic in Frontiers in Neuroscience, Frontiers in Physiology and Frontiers in Neurology, we have six original research articles that fit within the topic entitled “Stress-related diseases and dysfunctions”. The articles composing the topic are commented on according to their publication chronology.

The first article entitled “Heart Rate Variability Changes in Patients With Major Depressive Disorder: Related to Confounding Factors, Not to Symptom Severity?” from Sarlon et al. provided evidence-based on the complexity of the autonomic changes in humans with major depressive disorders (MDD). These authors reported that humans with MDD display alterations in the autonomic reactivity to acute stress exposure, as revealed by the association of symptom severity with higher sympathetic arousal in an emotion-driven imaginative and retrieval-generated stressor condition and a higher respiratory rate in the relaxed condition. Such correlation with symptoms was not detected for arterial pressure, thus supporting respiratory reactivity as a useful and easy-to-obtain index for autonomic function in patients diagnosed with MDD.

The second article “Both Prelimbic and Infralimbic Neurotransmissions Modulate Cardiovascular Responses to Restraint Stress in Rats” from Oliveira et al. reported on the role of adrenoreceptors expressed in the medial prefrontal cortex (mPFC) in the amplitude of the cardiovascular responses to an acute session of stress. Prelimbic (PL) and infralimbic (IL) are subregions of this cortical area implicated in physiological and behavioral responses to aversive threats. Although slightly different, the noradrenergic neurotransmission mediated by α-1 and α-2 adrenoreceptors in both PL and IL mPFC subregions plays a facilitatory role in cardiovascular and autonomic responses to restraint stress.

The third article authored by Gecaite-Stoncience et al. studied the fatigue and psychophysiological reactions in patients under high risk of heart attack. They investigated the links between cardiovascular responses to mental stress and fatigue in coronary arterial disease (CAD) patients after acute coronary syndrome (ACS). Findings showed that anticipatory cardiovascular reactivity to mental stress challenge was diminished in CAD just after ACS.

The manuscript “Maternal Separation Stress Affects Voluntary Ethanol Intake in a Sex Dependent Manner” by Bertagna et al. followed the hypothesis that maternal separation stress may increase ethanol consumption in adulthood in a sex-dependent manner, by provoking neuroadaptations in some encephalic areas. This study reported that neuronal activation patterns in basolateral and central amygdala subregions were modified and this was related to sex differences in rodents undergoing maternal separation stress.

Morais-Silva et al. in the article “Cardiovascular Reactivity to a Novel Stressor: Differences on Susceptible and Resilient Rats to Social Defeat Stress” used social interaction test (SIT) to identify a degree of susceptibility to produce exacerbated cardiovascular responses to social defeat stress (SDS) in rodents. By using the SDS exposure followed by the SIT, this study reported resting tachycardia and greater pressor responses to an acute session of restraint stress, a novel stress paradigm that evoked responses that contrasted to resilient–not susceptible–animals. They proposed that the increases in the magnitude of the cardiovascular reactivity to a novel stressful stimulus may be related to an augmented risk of developing cardiovascular diseases.

Stressful events in early life are known to produce several alterations in adulthood. Early maternal separation is a model of choice to evaluate the implication of stress in the pathophysiology of gastrointestinal diseases later in life. The report “Maternal separation induced visceral hypersensitivity evaluated via novel and small size distention balloon in post-weaning mice” by Enfu Tao et al. tested a novel and small size distention balloon methodology to assess irritable bowel syndrome in mice. These authors validated their small size distention balloon for evaluation of post-weaning visceral hypersensitivity induced by maternal separation stress.

We are grateful to the Frontiers in Neuroscience, Frontiers in Physiology and Frontiers in Neurology for supporting this Research Topic. The articles collected for the topic will extend the knowledge in the Autonomic Neuroscience and Integrative Physiology fields. This Research Topic adds novel information on the mechanisms potentially involved in the organization of responses to stress and on the pathophysiological processes, thus supporting therapeutic advances aimed at reducing stress-related morbidity and mortality.
